# Considerable Production of Ulvan from *Ulva lactuca* with Special Emphasis on Its Antimicrobial and Anti-fouling Properties

**DOI:** 10.1007/s12010-022-03867-y

**Published:** 2022-03-26

**Authors:** Mohamed I. A. Ibrahim, Mohamed S. Amer, Hassan A. H. Ibrahim, Eman H. Zaghloul

**Affiliations:** National Institute of Oceanography and Fisheries, NIOF, Cairo, Egypt

**Keywords:** *Ulva lactuca*, Ulvan, Sulfated polysaccharide, Antimicrobial activity, Anti-fouling

## Abstract

In the current study, a significant amount of ulvan was extracted from *Ulva lactuca *collected from Alexandria coastline, Egypt, using a simple extraction method. According to the chemical analysis, the obtained polysaccharide content is estimated to be 36.50 g/100 g with a high sulfate content of 19.72%. Physio-chemically, the FTIR analysis confirmed the presence of sulfated groups attached to the carbohydrate backbone. The GC–MS results revealed the presence of various monosaccharides with relative abundances in the order: fucopyranose (22.09%) > L-rhamnose (18.17%) > L-fucose (17.46%) > rhamnopyranose (14.29%) > mannopyranose (8.59%) > α-D-glactopyranose (7.64%) > galactopyranose (6.14%) > β-arabinopyranose (5.62%). In addition, the SEM–EDX depicted an amorphous architecture with a majority wt% for the elements of C, O, and S. The partially purified ulvan demonstrated potent antimicrobial activity against some fish and human pathogenic microbes. The inhibition zone diameter ranged from 11 to 18 mm. On the other hand, the prepared ulvan-chitosan hydrogel significantly improved the antimicrobial activity as the inhibition zone diameter ranged from 12 to 20. Moreover, when compared to the controls, the extracted ulvan demonstrated anti-fouling properties and successfully disrupted the biofilm formed on a glass slide submerged in seawater.

**Supplementary Information:**

The online version contains supplementary material available at 10.1007/s12010-022-03867-y.

## Introduction

Sulfate polysaccharides (SP) derived from marine algae such as fucoidan and ulvan have received significant attention as potential biomaterials. The SP are unique since they combine the chemical diversity and biocompatibility of polysaccharides with unrivaled bioactivities such as antimicrobial, anti-fouling, antioxidant, anticancer, and anti-coagulant that are not found in any other chemical compounds [1]. The potential of different SP has been demonstrated as antimicrobial agents against a wide range of human and fish pathogens [2]. The utilization of fucoidan has some drawbacks associated with its complex and heterogeneous structure. In this regard, the SP ulvan has drawn the attention of many researchers, as it mainly contains uronic acid and sulfate groups [3]. The use of ulvan is advantageous in bioactivity and availability as it is extracted from the *Ulva* species, particularly *U. lactuca*, which constitutes the most abundant algal biomass [4].

Ulvan is a cell wall polysaccharide that accounts for 9 to 36% dry weight of the biomass of *Ulva* species. It is mainly composed of sulfated rhamnose uronic acids (glucuronic acid and iduronic acid) linked via a 1,4-glycosidic bond [5]. Moreover, ulvan has been utilized in coating processes, and its ability to inhibit both Gram-positive and Gram-negative bacterial adhesion on surfaces has been reported [6]. The extraction and purification procedures applied to obtain the SP from marine algae considerably affect its yield, chemical structure, and biological activity [7]. Furthermore, the bioactivity of ulvan was largely dependent on its molecular weight, uronic acid concentration, and the presence of sulfate groups [8, 9]. The adhesion resistance to diverse fouling organisms has not been thoroughly investigated despite the outstanding performance [10].

Marine fouling on artificial surfaces has long been a problem in the marine industry, as excessive adhesion of marine foulants on the surface of ships eventually leads to increased fuel consumption. As a result, the development of natural, biocompatible new materials for controlling marine fouling is required. Even though the fundamental concepts of preventing bacterial adhesion and marine fouling are very similar, the use of ulvan is thought to contribute to marine fouling control [11].

Therefore, the current investigation proposed extracting ulvan, as a potent sulfated polysaccharide, from *U.* *lactuca* in significant quantities following a simple and quick method. It was chemically analyzed, and its antimicrobial effectiveness was evaluated against both fish and human pathogens, besides its potential anti-fouling properties. Moreover, it was chemically modified by combining it with chitosan to increase its antimicrobial activity further and compare it to commercial antibiotics.

## Material and Methods

### Collection and Identification of Green Algae

**Fig. 1:**
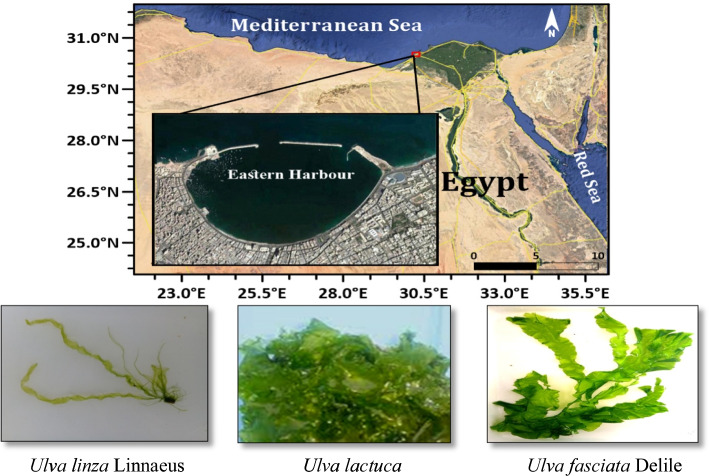
Sites of algal sample collection for ulvan extraction

Several green algae samples (*U. linza* Linnaeus, *U. lactuca*, and *U. fasciata* Delile) were collected from different sites in the marine environment along Alexandria coastline, especially at Eastern Harbor, Egypt, as shown in Fig. 1. Preliminarily, the algae were washed well with tap water and then dried in the open air for 72 h. Finally, they were cut into 2-cm pieces. The collected green algae were identified according to taxonomical reference guides, and the investigated algae were microscopically identified according to Braune [12].

### Reference Microbes and Culture Media

Six Gram-positive bacterial pathogens (*Streptococcus agalactiae*,* Staphylococcus aureus* ATCC 25,923, *Enterococcus faecalis* ATCC 29,212, *Bacillus Subtilis* ATCC 6633, *Staphylococcus epidermidis*, and *Listeria monocytogenes* ATCC 35,152), as well as six Gram-negative bacterial strains (*Aeromonas hydrophila*,* Pseudomonas fluorescens*,* Pseudomonas aeruginosa* ATCC 9027, *Escherichia coli* ATCC 8739,* Klebsiella pneumonia* ATCC 13,883, and *Bordetella pertussis* ATCC 8467), were used as reference strains. In addition, three fungal strains (*Aspergillus niger*,* Penicillium notatum*, and *Fusarium solani*) and one yeast strain, *Candida albicans* ATCC 10,231, were used. The Microbiology Laboratory provided the strains (National Institute of Oceanography and Fisheries, Alexandria, Egypt). Four media were used to cultivate the reference strains and to detect the antimicrobial activity of ulvan [13]. They were nutrient broth (NB) (Oxoid, USA) and nutrient agar (NA) (Oxoid, USA) for bacteria and yeast, potato dextrose broth (PDB) (HiMedia, India), and potato dextrose agar (PDA) (HiMedia, India) for fungi.

### Extraction and Purification of Crude Ulvan

Since it is more abundant than the others, *U. lactuca* was chosen to carry out the remainder of the study (*U. linza* Linnaeus and *U. fasciata* Delile). In order to obtain a significant amount of ulvan, the De Jesus Raposo et al. [14] method was followed with significant changes to obtain considerable quantity of ulvan. Briefly, the mature seaweeds from *Ulva lactuca *were dried at 105 °C for 3 h, ground, and sieved through 80 mesh screens. The seaweed powder was de-pigmented with 100 mL hexane under shaking at 3000 rpm for 24 h, followed by filtration. The residue of the samples was soaked in 120 mL ethanol (95%) for 24 h at room temperature with gentle shaking to remove soluble materials and undesirable impurities, including free sugars, amino acids, some phenols, and low molecular weight compounds [15]. After filtration, the residue was washed with ethanol, dried under vacuum at 60 °C for 3 h, then stored in a plastic bag for the proximate analyses (protein, moisture, ash, fibers, and lipid) the recommended AOAC methods [16]. The partial purified SP was obtained following the method of Peasura et al. [17].

In brief, the dried crude ulvan powder (20 g) from the previous step was homogenized and refluxed with distilled water (1: 20, w/v) for 20 h at 85–90 °C. The extracted residue was separated by filtration through a cheesecloth, and the collected syrup was filtered through filter paper (Whatman No. 1). Afterward, the filtrate was concentrated by evaporating the water under vacuum at 40 °C and 77 mbar. The concentrated residue was allowed to cool and precipitate by adding fourfold volumes of cold ethanol (95%). The mixture was kept overnight, and the formed precipitate was collected by centrifugation at 12,000 rpm at 10 °C, washed twice with absolute ethanol, and then dried at 50 °C under vacuum overnight to obtain a polysaccharide. The partially purified SP sample was stored at 4 °C and used for further analysis [17].

### Chemical Composition of Crude and Partially Purified Ulvan

The humidity, ash, organic matter, lipid content, and the fibers in the crude ulvan were estimated according to AOAC (1990) and Pádua et al. [16, 18], while the nitrogen and protein were estimated according to Yokoyama and Guimarães [19]. Moreover, the carbohydrate content in the crude ulvan was calculated using the formula: Carbohydrate (%) = 100 − (percentage of humidity, protein, lipids, moisture, ash, and fibers) [17]. In contrast, the yield (%) of the SP in the partially purified ulvan was computed using the equation: SP $\left(\%\right)= (\frac{\mathrm{weight\;of\;extracted\;powder}}{\mathrm{weight\;of\;dry\;seaweed}}) \times 100$ [17, 20]. The total sugar content (carbohydrate as glucose) in the partially purified ulvan was determined according to the phenol–sulfuric acid method [21], using glucose as the standard. The results were calculated based on a glucose standard curve and expressed as grams per 100 g dried weight of the sample. Further, total sulfate percentage was determined by following the modified BaCl_2_ turbidimetric method [2, 22]. Acid hydrolysis was performed on 50 mg of partially purified SP samples. The total sulfate content as 100 g dried weight was quantified based on a sodium sulfate standard curve prepared from a series of concentrations from Na_2_SO_4_ upon turbidity formation by adding BaCl_2_ [23].

### Characterization of Partially Purified Ulvan from *U. lactuca*

#### Structural Characterization by Fourier Transform Infrared (FTIR) Spectroscopy

The functional groups in the extracted ulvan were identified using an FTIR spectrometer (Bruker, ALPHA, Germany) equipped with the attenuated total reflectance (ATR) technique. The dried sample (~ 2 mg) was loaded over a trough plate comprising a single germanium crystal. The spectra were acquired in the 4000–400 cm^−1^ range with a resolution of 4.0 cm^−1^ over 128 scans after subtracting the atmospheric background interferences [24].

#### Characterization by Nuclear Magnetic Resonance (NMR) Spectroscopy

Identification of the characteristic signals of the ulvan from *U. lactuca* was achieved through the ^1^H NMR experiment. Approximately 5 mg sample of the extracted ulvan was dissolved in 0.5 mL of deuterium oxide (D_2_O 99.9%; δ = 4.78 ppm) and transferred to an NMR tube [25, 26]. The NMR spectra were recorded using Bruker AvanceII NMR spectrophotometer (300 MHz) at 298 K, and the chemical shifts were expressed in parts per million (ppm, δ) relative to tetramethylsilane (TMS) as an internal standard.

#### Monosaccharide Identification by Gas Chromatography-Mass Spectrometry (GC–MS)

The monosaccharides of the extracted ulvan from *U. lactuca* were identified by converting the polysaccharide into the simple sugars’ derivatives before GC–MS analysis [26–29]. About 20 mg from the polysaccharide was transferred to a glass tube and subjected to acid hydrolysis using 2 mol/L sulfuric acid at 105 °C for 10 h. The tube was left to cool down, and the hydrolysates were neutralized with barium carbonate to pH 7.0. The precipitate was removed by centrifugation, while the supernatant was filtered through a 0.20-μm syringe filter and lyophilized, and then the dried sample was dissolved in 50 μL methanol. A silylating mixture of pyridine-hexamethyldisilazane-trimethylchlorosilane (9:3:1 v/v/v) was added using 50 μL per mg of dried sample. About 2 μL from the methyl-silyl sugar derivatives were injected into GC–MS (MassHunter 1989–2014, Agilent Technologies, Inc.). The separation and detection of the formed silyl sugar derivatives were accomplished using a previously reported method: the column used was HP5MS (30 m × 0.25 mm × 0.25 μm), the temperature of the detector and the injector were set at 320 °C, the column temperature was firstly set at 100 °C for 1 min, and then ramped from 100 to 260 °C at 4 °C for 1 min, and then the temperature was set for 10 min at 260 °C. Helium was used as carrier gas at 1 mL min^−1^ [30]. The detected sugars were identified by comparing their masses with the NIST library [31].

#### Method Validation

Linearity, lower detection limit (LOD), quantitation limit (LOQ), and accuracy were determined to validate the GC–MS method. Stock solutions were prepared for each derivatized monosaccharide standard at final concentrations of 7.30, 1.85, 1.68, 3.69, 3.72, 2.22, 2.24, and 2.22 mg/ml for fructose, xylose, arabinose, rhamnose, fucose, glucose, mannose, and galactose, respectively. Serial diluted concentrations from each standard were prepared to construct the calibration curves between the recorded areas *versus* concentrations. Determination of the LOD and LOQ was done at signal-to-noise ratio of 0.3. The accuracy was validated by calculating the recovery (R%) of each monosaccharide through spiking reference samples of known sugars’ concentrations with accurate amounts of standards of known concentrations. Then, $R\%= \frac{\left(Total\;concentration-Reference\;concentraion\right)}{Spiked\;concentration } x 100$, which reflected an accepted range of recovery (89–113%).

#### Morphological and Elemental Analyses by SEM and EDX Spectroscopy

In order to gain insight into the morphology and surface texture, the partially purified ulvan from *U. lactuca* was studied using high-resolution scanning electron microscopy (HRSEM; JSM-IT 200, Jeol, Japan) under high vacuum, acceleration voltage of 15 kV, and large field detector. The sample for SEM analysis was prepared by coating with gold (15°A) for 2 min by physical vapor deposition [24, 28]. Additionally, the quantitative elemental composition of the polysaccharide was analyzed using a scanning electron microscope–energy dispersive X-ray (SEM–EDX) spectrometer. No pretreatment was performed for the EDX measurement. The weight and atomic percentages of the different elements in the sample were related to the emitted X-rays [32].

#### Size Exclusion Chromatography (SEC)

The average ($\overline{Mw }$) of the different polysaccharides of the ulvan from *U. lactuca *was estimated by size exclusion chromatography (SEC) using DMSO/NaNO_3_ (8.5 g/L) as eluent at 70 °C. The sample was prepared at a concentration of 10 mg/mL and then filtered through 0.45 µm prior the injection of 20 µL. The separation and detection were achieved using three PL gel columns (100,000, 1000, and 100 Å), and the multi-angle laser light scattering detector (MALLS, Mini-Dawn®, Wyatt, Milford, USA). The average ($\overline{Mw }$) for the polysaccharides were estimated using a calibration curve constructed from a range of dextran’s standards (6.0–470 kDa) and then applying the deconvolution method using origin 2022.

### The Antimicrobial Activity of the Extracted Ulvan from *U. lactuca*

The well-cut diffusion technique was used to evaluate the antimicrobial activity of the extracted ulvan against the reference bacteria, yeast, and fungal strains. A volume of 15 mL of sterilized NA for bacteria and yeast was prepared in caped test tubes and allowed to cool to 50 °C in a water bath. A 0.5 ml inoculum (10^8^ CFU for bacteria and yeast) was added. The tubes were mixed using a vortex for 15–30 s. Subsequently, the contents of each test tube were poured into a sterile Petri dish for solidification [33]. The activity was evaluated using the well-cut diffusion technique, in which wells were punched out with a sterile 0.7-cm cork-borer in the agar plates containing the tested microorganisms. About 100 µL of extracted ulvan were added to each well. They were subjected to 4 °C incubation for 2 h to allow diffusion and then incubated at appropriate temperatures for 24 h. The results were obtained by measuring the diameter of the inhibition zone around each well and expressed in millimeters (mm). The experiment was conducted in triplicates, and data were represented as means ± standard deviation (SD) [34]. On the contrary, ulvan was also tested against the indicator fungi by placing one disc of the fungal strain on the top of PDA plates. As previously stated, all plates were incubated at 30 °C, and the inhibition zones were observed [35].

### The Effect of Autoclaving on the Ulvan Antimicrobial Activity

Its susceptibility to autoclaving was investigated. In summary, ulvan was subjected to autoclaving (121 °C, 1.5 Pa) for 15 and 30 min to confirm the nature and activity of the extracted ulvan, and then its antimicrobial activity was detected, as mentioned early.

### Preparation of Ulvan-Chitosan (UC) Hydrogel

For the preparation of the hydrogel, the method described by Gobinath et al. [36] was followed. Chitosan was dissolved in 1% glacial acetic acid, and then 1 mL of deacetylated chitosan solution (40 mg/mL) was mixed with 1 mL of ulvan (10 mg/mL) and then incubated at 50 °C for 10 min to get the UC hydrogel.

### The Minimal Inhibitory Concentration of Ulvan and UC Hydrogel

The minimum inhibitory concentrations (MIC) of the extracted ulvan and prepared UC hydrogel against the tested pathogens were determined according to the broth micro-dilution method guided by the Clinical and Laboratory Standards Institute [37], with slight modification. All samples were tested at the final concentrations of 0.0–50 mg mL^−1^ in LB broth, and the tested bacterial cell concentration was 10^5^ CFU/mL. The incubation was conducted at appropriate temperatures for 24 h. The MIC was read as the lowest concentration of the tested sample that inhibited visible pathogen growth. Negative control was prepared containing LB medium only.

### Antibiotic Susceptibility Test for Bacterial Pathogens

Antibiotic susceptibility was determined as described by Syal et al. [38] to compare the bioactivity of extracted ulvan and its hydrogel to standard antibiotics. Briefly, overnight cultures of tested pathogens were inoculated in nutrient agar plates; antibiotic discs (Oxoid, England) were added to the surface of the agar and incubated for 24 h at appropriate temperatures. The susceptibility was detected by measuring the inhibition zone diameter around each disc.

### The Anti-biofouling Activity of the Extracted Ulvan

For 24 h, 1 mL of fouling bacteria-containing seawater was incubated with glass slides in 2-L conical flasks containing 1 L of sterilized seawater at 30 °C. The extracted ulvan was added to the flasks in a concentration of 1000 mg/L as an anti-fouling agent. However, a control flask without ulvan was used for comparison [39]. After dying with 0.4% crystal violet solution for 10 min, the glass slide was washed with distilled water, air-dried, and examined under a light microscope (model BXFM-S, Olympus, Tokyo, Japan).

### Statistical Analysis

Data are expressed as mean ± SD of three independent experiments. It was analyzed by one-way analysis of variance (ANOVA) using SPSS17.0 software at the significance level of *P* < 0.05.

## Results and Discussion

### Purification and Characterization

Bioactive polysaccharides that contain sulfate groups, exclusively obtained from algal resources, are gaining increasing interest as raw materials for several applications. The prominent representatives of sulfated polysaccharides are ulvan from green seaweed, fucoidan from brown seaweed, and carrageenan from red seaweeds. Chemically, the structures of these polysaccharides were found to vary according to the algal origin and extraction procedures [40]. Therefore, the current study was carried out to extract ulvan from the green algae, *U. lactuca*, collected from Alexandria coastline, as well as analyze its chemical structure and potential biological activity.

The proximate chemical analyses of the crude and partially purified ulvan from *U.* *lactuca* are summarized in Table 1. The dried crude ulvan contains excessive amounts of ash (39.29%) and carbohydrates of 24.27%. The high carbohydrate content and lower protein content (9.67%) may relate to increasing the photosynthetic activity of the seaweeds during the collection period (July), which increases their growth rate and maturity [41, 42]. Otherwise, the high ash content was mainly due to the high sulfate content in seaweed associated with the maximum growth period [43], which is consistent with other reported studies [17, 26].

**Table 1:** Proximate chemical analysis of both ulvan crude and the extracted sulfated polysaccharide from *U. lactuca*

Parameter	Value $\pm$ SE
	(%)
Crude ulvan	
Moisture	13.98 $\pm$ 0.756
Ash	39.29 $\pm$ 0.430
Fibers	3.22 $\pm$ 0.292
Carbohydrates	24.27 $\pm$ 0.471
Proteins	9.67 $\pm$ 0.292
Total lipids	9.57 $\pm$ 1.192
Extracted ulvan (partially purified)	
Carbohydrates as glucose	43.61 $\pm$ 0.014
Sulfated polysaccharides	36.5 $\pm$ 0.346
Sulfate	19.72 $\pm$ 0.212

In this study, the water extraction method for the crude ulvan from *U. lactuca* yielded approximately 36.5% of the sulfated polysaccharide, which is higher than the yield produced from *Ulva fasciata* (16.28%) [20] and *Ulva ohnoi* (14.84%) [44]. The UV–Vis analysis for the extracted ulvan powder revealed a carbohydrate content of 43.61%, which is higher than the content produced from *Ulva lactuca* (27.41%) and *Gracilaria gracilis* (33.52%) [31]. Indeed, the sulfate within the seaweed cell walls plays a vital role by allowing the plant to survive under environmental stress such as saline ecosystems [17]. In the current study, the sulfate content of the extracted polysaccharide from *U.*lactuca was 19.72% which is similar to the sulfate content in ulvan produced from Ulva linza (17.2%) [45] and Ulva pertusa (19.7%) [2], within the range (16.0–26.8%) of ulvan from *Ulva lactuca* [46], higher than ulvan from *Ulva armoricana* (10.3–13.8%) and *Ulva rotundata* (9.2–12.5%) [47, 48], lower than ulvan from *Ulva intestinalis *(34–40%) [17], and *Ulva conglobata* (23.04–35.20%) [26]. This finding can be attributed to the difference in the percentages of the chemical composition of the cell walls between Ulva species.

**Fig. 2:**
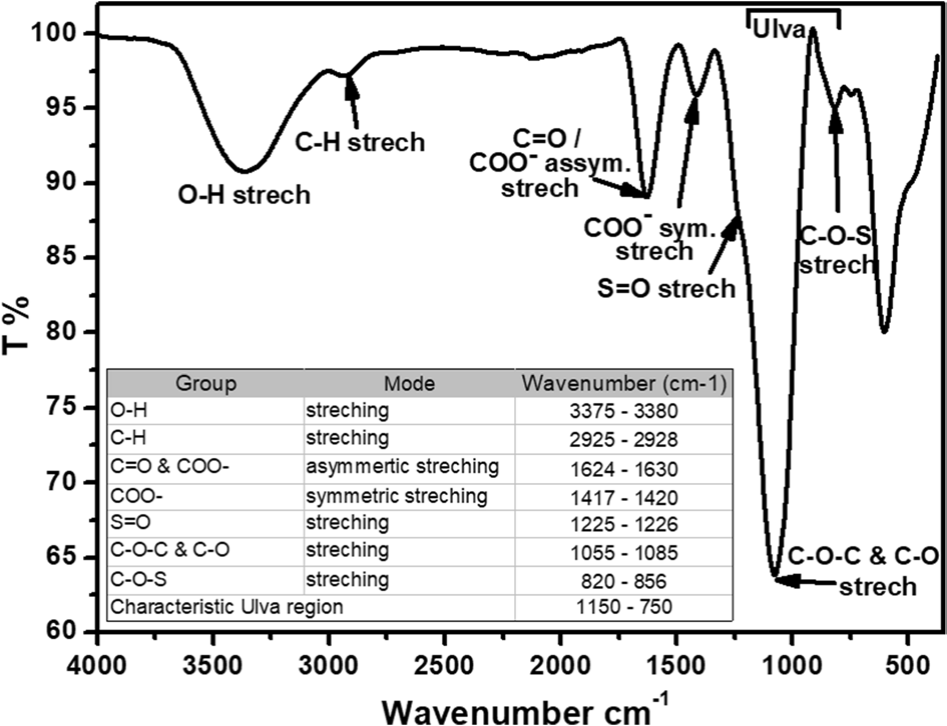
FTIR spectrum of the partial purified ulvan from *U. lactuca*

The FTIR analysis of the partially purified ulvan showed several characteristic peaks of sulfated polysaccharides. The spectrum is identical and comparable to the IR spectra of the sulfated polysaccharides obtained from other *Ulva* species [17, 25, 31, 46, 49]. Subsequently, the assignment of the IR bands was based on the published data of the sulfated polysaccharides, and the data are summarized in Fig. 2. The FTIR spectrum showed a broad and strong absorption band at 3375–3380 cm^−1^, corresponding to the stretching vibration of the hydroxyl (O–H) group in the polysaccharide structure. The weak shoulder at 2925–2928 cm^−1^ was related to the stretching vibration of the aliphatic C-H bond of the methyl group, which is characteristic of polysaccharides [26]. The signal around (1624–1630 cm^−1^) was assigned to the stretching vibration of the (C = O) group and asymmetric stretching vibration of the (COO-) group, while the signal around (1417–1420 cm^−1^) was allocated to the symmetric stretching vibration of the COOH group’s bond [26, 46, 47, 50]. The sulfated nature of the polysaccharide was ascertained by the absorption band in the region 1225–1226 cm^−1^, which is related to stretching vibration of the sulfate ester (S = O) group, and by the shoulder at 820–856 cm^−1^ [8, 25, 31, 46, 51], which is assigned to the C-O-S stretching of axial sulfate groups as reported for ulvan from *U. rigida* [52]. Furthermore, the region between 1150 and 750 cm^−1^ has been reported as a typical signature to ulvans [53]. Finally, the band at 1055–1085 cm^−1^ was assigned to the stretching vibration of the C–O–C band [46].

### Structural Analysis of the Extracted Ulvan by ^1^H NMR

**Fig. 3:**
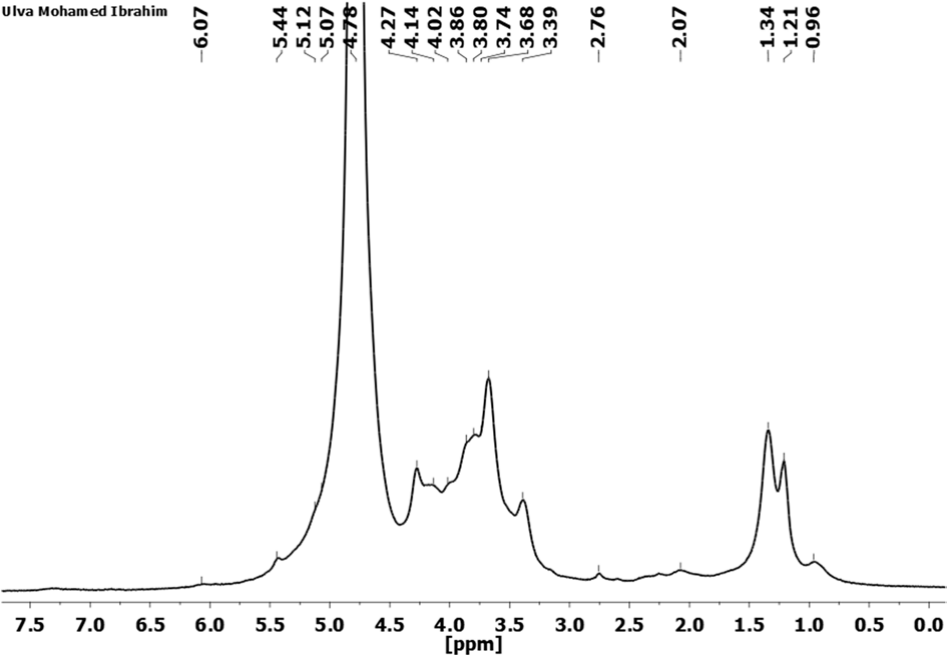
^1^H NMR spectra of the extracted ulvan from *U. lactuca*

Various *Ulva* species have been identified as a rich source for the sulfated polysaccharides composed of rhamnose, xylose, glucose, galactose, mannose, arabinose, and rich content of sulfate [26, 31, 49]. It has been reported that the sulfated polysaccharides revealed different molar ratios of sugars’ composition, which was attributed mainly to the extraction methods [47, 54–56]. The sulfated polysaccharides possess complex and heterogeneous structures, and the sulfate groups interrupt the interpretation of the connectivity and branching from the NMR analysis. Additionally, broadening and overlapping peaks within the ^1^H NMR spectrum limit the chemical description of the extracted Ulva structure (Fig. 3) [8]. However, the current study’s proton signal assignment was based on the chemical shifts reported for ulvans in the literature [5, 43, 48, 57, 58]. As previously reported, the region 3.2–5.4 ppm showed overlapped signals representative of the hybrid nature of polysaccharides probably composed of rhamnose, xylose, glucose, and galactose [48].

The two intense peaks at 1.21 and 1.34 are assigned to the protons of the methyl groups of non-sulfated and sulfated α-L-rhamnosyl residues, respectively [26, 49, 59]. The chemical shifts emphasized the sulfated nature of the -L-rhamnose at 1.34 and 5.44 ppm, as previously reported for *U. clathrate* [25]. The anomeric signals in the region 4.5–5.4 ppm were close to that observed for ulvan in previous studies corresponding to the α- and β-types of the glycosidic bonds [46]. Nevertheless, assigning all of the protons in this region has proven difficult since the signals are mainly superimposed by the deuterium oxide (D2O; 4.78 ppm) peak [49].

### Monosaccharides’ Composition of the Extracted Ulvan by GC–MS

**Fig. 4:**
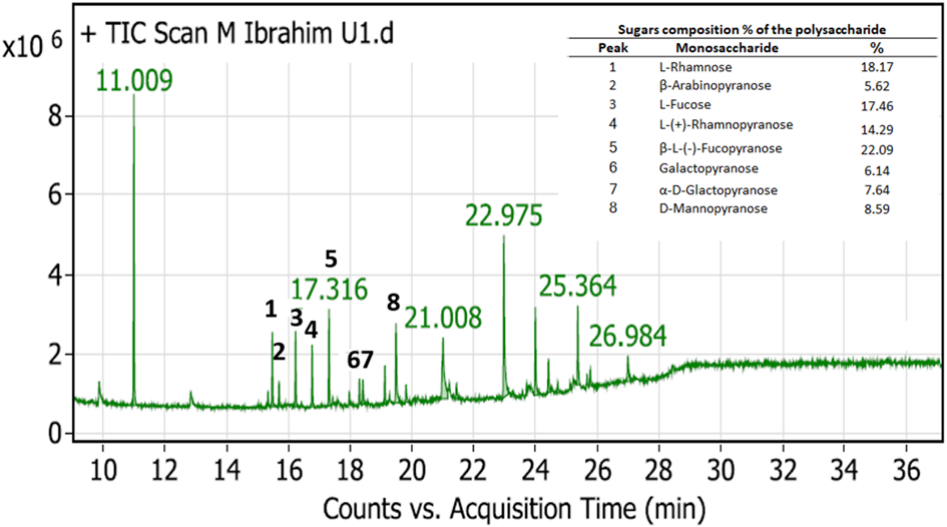
GC chromatogram representing the relative sugar compositions (%) for the partial purified ulvan from *U. lactuca*

Ulvan is a polysaccharide whose heterogeneous composition varies depending on the algal biomass’ taxonomic origin and harvesting season [5]. It was challenging to accurately define the composition of the extracted ulvan, which may be attributed to the complexity of its structure and the presence of several types of sugars. Acid hydrolysis is the most effective method of depolymerizing the polysaccharides into their monomeric units, followed by identification using the appropriate chromatographic technique [43]. In the current study, the sulfated polysaccharide from *U. lactuca* was found to be primarily composed of rhamnose and fucose, with lower amounts of galactose, arabinose, and mannose. The GC–MS spectrum of the extracted ulvan and the sugar composition (%) are demonstrated in Fig. 4. The results revealed that the relative sugars% are as follows: fucopyranose (22.09%) > L-rhamnose (18.17%) > L-fucose (17.46%) > rhamnopyranose (14.29%) > mannopyranose (8.59%) > α-D-glactopyranose (7.64%) > galactopyranose (6.14%) > β-arabinopyranose (5.62%).

The sugar compositions were similar to those found in ulvan obtained from other studies. Masakuni et al. [2] reported the molar ratios (4.0: 0.1: 0.3) for L-rhamnose, D-xylose, and D-glucose residues, respectively, for the ulvan extracted from *U. pertusa*. Van Tran et al. [60] found that the ulvan from *U. reticulata* is composed of rhamnose, galactose, xylose, mannose, and glucose with a molar ratio of 1:0.12:0.1:0.06:0.03. Also, our results are compatible with those obtained by Olasehinde et al. [31], who reported that ulvan from *G. gracilis* and *U. lactuca* is composed of rhamnose and galactose ribose, arabinose, glucose, xylose, and mannose with different sugar compositions. Moreover, the study is similar to the monosaccharides of the ulvan from marine green algae *U. conglobate* [26]. They reported that it is composed of rhamnose, glucose, xylose, fucose, galactose, mannose, and arabinose with higher contents of rhamnose > 60% and glucose > 13%, while the other saccharides revealed minor contents [26]. Additionally, the ulvan from *U. intestinalis* demonstrated the composition of rhamnose (12.70–39.24%) and glucose (0.84–11.86%) as major constituents, which is similar to the current study of *U. lactuca *[17].

### Morphological and Elemental Studies by SEM and EDX Spectroscopy

**Fig. 5:**
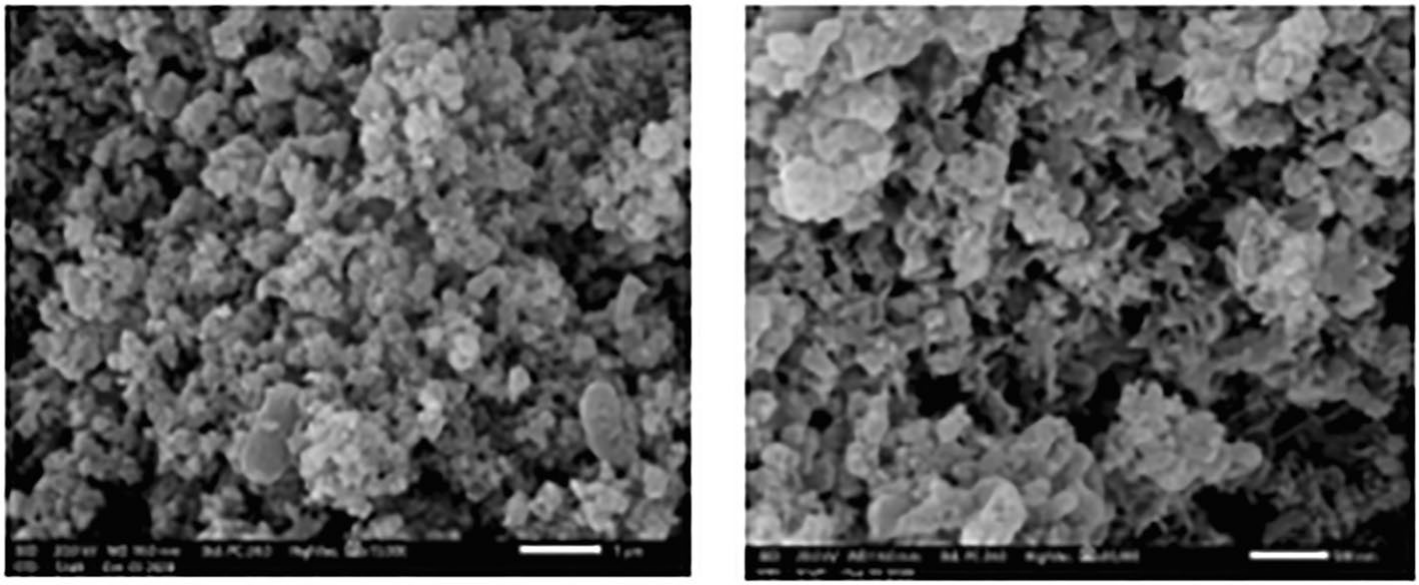
SEM micrographs of the extracted ulvan from *U. lactuca*

**Fig. 6:**
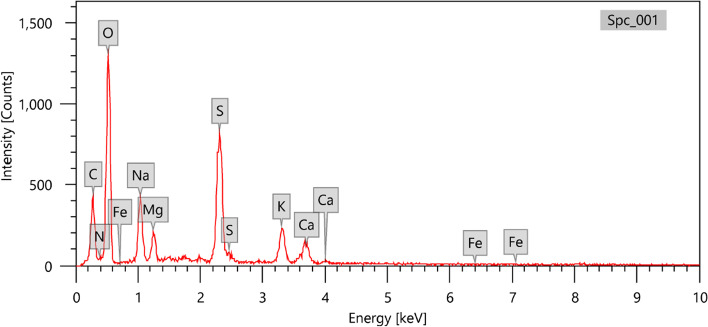
SEM–EDX data of the extracted ulvan from *U. lactuca*

The SEM micrographs of the semi-pure ulvan demonstrated an amorphous non-smooth texture (Fig. 5). Interestingly, the EDX analysis highlighted the polysaccharide’s sulfated nature (Fig. 6 and Table 2). The EDX indicated the presence of different elements, particularly oxygen, carbon, sulfur, sodium, potassium, magnesium, calcium, and nitrogen, of 48.87, 26.92, 9.11, 6.18, 3.30, 2.09, 2.00, and 1.48%, respectively. These findings revealed that the polysaccharide contains sulfuryl groups as determined by UV and FTIR analyses, confirming its sulfated nature with the presence of a protein consistent. The findings support the theory that ulvan is a sulfated polysaccharide present in the algal cell wall, associated with proteins [5, 58, 61].

**Table 2:** Average mass values (%) for the elemental analysis of the ulvan polysaccharide from *U. lactuca*

Element	*Mass (%)
C	26.92 ± 0.30
O	48.87 ± 0.51
N	1.48 ± 0.21
S	9.11 ± 0.13
Na	6.18 ± 0.15
K	3.30 ± 0.10
Mg	2.09 ± 0.08
Ca	2.00 ± 0.08
Fe	0.05 ± 0.03

*Mean values of three replicate readings for the ulvan polysaccharide from *U. lactuca*

The EDX analysis demonstrated the nitrogen content of 1.48%, accounting for 9.25% using a multiplication factor of 6.25 [62], and this is consistent with the proximate analysis of the extracted ulvan (9.67%). The protein content is comparable or lower than those described in the literature. Hence, the protein levels were 7.1–22% for ulvan isolated from *U. rotundata* and 10.9–16.8% for *Ulva armoricana* [63]. Also, the estimated protein content in the present study (Egypt) was higher than the protein content extracted from *Ulva lactuca* (Tunisia) (3.54%) [20], *Ulva lactuca* (France) (6.25%), and *Ulva clathrata* (0.39%) [25].

Moreover, the sulfur content in the current study (9.11%) was similar to that obtained from *U. lactuca* (PULV) 9.67% [31]. The carbon content was close to that recorded for the purified fucoidan (26.12%) of *F. vesiculosus* [64], while lower than reported for the *Gracilaria gracilis* (PGCL: 29.49%) [31]. Na, K, Mg, and Ca were also reported for polysaccharides from *Gracilaria gracilis* (PGCL) and *U. lactuca* (PULV) with comparable amounts [31].

### Size Exclusion Chromatography (SEC)

**Fig. 7:**
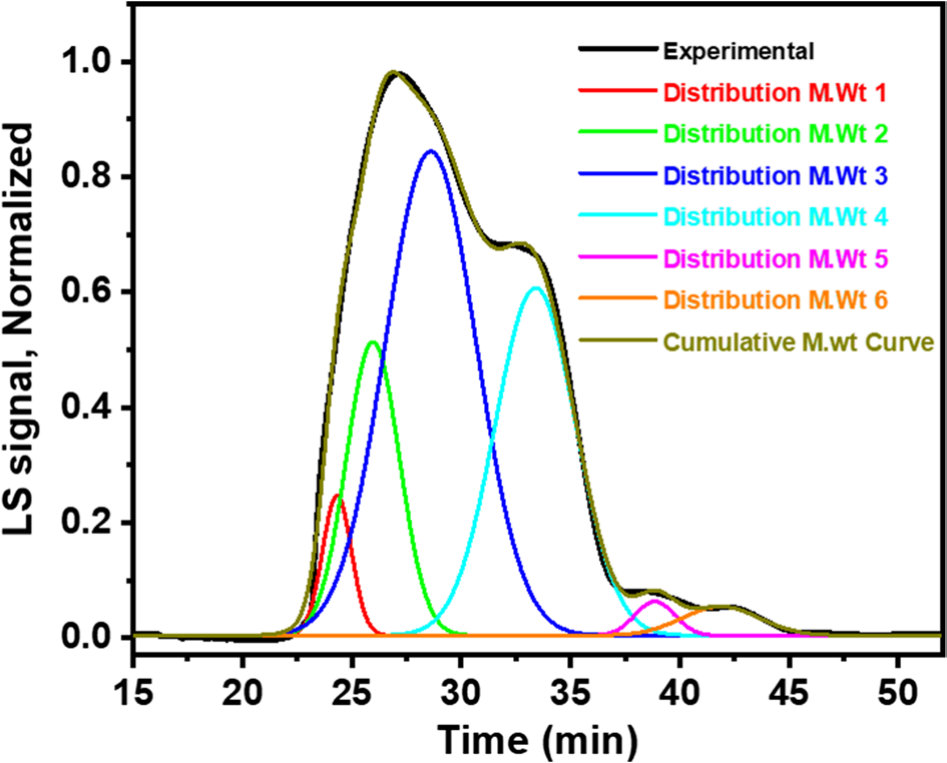
Deconvoluted Gaussian bands for the SEC chromatogram of the ulvan from *U. lactuca*

Ulvan from *U. lactuca *showed a mixture of polysaccharides of a wide range of molecular weights ($\overline{Mw }=$ 1.81 – 125 kDa) (Supplementary Table S1 and Fig. 7). Applying the deconvolution method could provide six Gaussian curves of different $\overline{Mw }$ with a good fitting (R^2^ = 0.999) (Supplementary Table S2 and Fig. 7). Three dominant Gaussian distributions were distinguished with highest areas of 46.12%, 19.50, and 16.35% representing $\overline{Mw }$ of 44.3, 14.0, and 83.9 kDa, respectively, using the calibration curve equation of dextran standards ($\mathrm{Log M}.\mathrm{wt}= -0.1047\mathrm{ RT}+4.6408$; R^2^ = 0.955). The SEC curve revealed overlapped peaks similar to that obtained for the polysaccharides from *U. intestinalis*; however, the $\overline{Mw }$ in the current study are less than that reported for *Ulva intestinalis* for water extraction (300 kDa), and 0.1 N sodium hydroxide extraction (110 kDa), while close to the polysaccharides of 0.1 N hydrochloric acid extraction (88 kDa) [17]. The $\overline{Mw }$ variations are mainly related to involved species as well as the experimental conditions for ulvan production.

### The Antimicrobial Activity of the Extracted Ulvan from *U. lactuca*

**Fig. 8:**
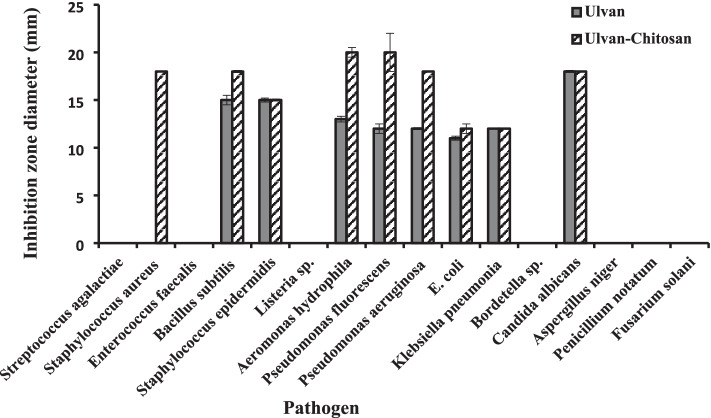
Antimicrobial activity of extracted ulvan and prepared ulvan-chitosan hydrogel against different indicator pathogens

The partial purified ulvan extracted from *U. lactuca *showed clear antimicrobial activity against Gram-positive and Gram-negative bacteria; most are known as fish and human pathogens besides the pathogenic yeast *C. albicans*. Collective data in Table 3 and Fig. 8 indicate that the values of inhibition zones ranged between 11 mm against *E. coli* ATCC 8739 and 18 mm against *C. albicans* ATCC 10,231. However, ulvan did not demonstrate any activity against two Gram-positive bacterial pathogens (*S. aureus* ATCC 25,923 and *L. monocytogenes* ATCC 35,152), Gram-negative bacterial pathogen (*B. pertussis*. ATCC 8467), and fungal pathogens tested (*A. niger*, *P. notatum*, and *F. solani*).

**Table 3:** Antimicrobial activity, and MICs, of extracted ulvan and prepared ulvan-chitosan hydrogel against different indicator pathogens

Pathogen	Inhibition zone diameter (mm)		MIC (mg/mL)	
	Ulvan	Ulvan-chitosan hydrogel	Ulvan	Ulvan-chitosan hydrogel
Gram-positive strain				
^ *^S. agalactiae	-	-	-	-
* S. aureus* ATCC 25,923	-	18 ± 0.05	-	12.5 ± 0.0
* Ent. faecalis* ATCC 29,212	-	-	-	-
* B. subtilis* ATCC 6633	15 ± 0.50	18 ± 0.11	12.50 ± 0.0	3.12 ± 0.0
^ *^S. epidermidis	15 ± 0.21	15 ± 0.11	6.25 ± 0.0	0.78 ± 0.0
* L. monocytogenes* ATCC 35,152	-	-	-	-
Gram-negative strain				
^ *^A. hydrophila	13 ± 0.30	20 ± 0.50	12.50 ± 0.0	1.56 ± 0.0
^ *^P. fluorescens ATCC 17,386	12 ± 0.50	20 ± 2.00	12.50 ± 0.0	3.12 ± 0.0
* P. aeruginosa* ATCC 9027	12 ± 0.10	18 ± 0.05	25.00 ± 0.0	3.12 ± 0.0
* E. coli* ATCC 8739	11 ± 0.21	12 ± 0.50	6.25 ± 0.0	0.78 ± 0.0
* K. pneumoniae* ATCC 13,883	12 ± 0.00	12 ± 0.05	6.25 ± 0.0	0.78 ± 0.0
* B. pertussis* ATCC 8467	-	-	-	-
Yeast strain				
* C. albicans* ATCC 10,231	18 ± 0.10	18 ± 0.05	12.5 ± 0.0	3.12 ± 0.0
Fungus strain				
* A. niger*	-	-	-	-
* P. notatum*	-	-	-	-
* F. solani*	-	-	-	-

^*^Indicate fish pathogens.

Based on the data depicted in Table 3 and Fig. 8, the UC hydrogel showed apparent activity against *S. aureus* ATCC 25,923 (18 mm). In addition, the prepared hydrogel demonstrated significant improvement in antimicrobial activity since it is increased from 12 and 15 mm to 18 mm against *P. aeruginosa* ATCC 9027 and *B. subtilis* ATCC 6633, respectively. Moreover, the hydrogel raised the antibacterial activity from 12 and 13 mm to 20 mm against both *A. hydrophila* and *P. fluorescens* ATCC 17,386, respectively. The hydrogel had the same activity against *C. albicans* ATCC 10,231 as ulvan (18 mm). Neither UC hydrogel nor ulvan demonstrated antifungal activity.

The MIC values for both the prepared ulvan and UC hydrogel were evaluated. Data in Table 3 indicate that the MIC values for ulvan ranged from 6.25 to 25 mg/mL, while the prepared UC hydrogel significantly had lower MIC values ranging from 0.78 to 3.12 mg/mL except for *S. aureus* ATCC 25,923 that showed MIC of 12.5 mg/mL. On the contrary, ulvan did not show any activity against it at any tested concentration, which means to exert antimicrobial activity, higher concentrations are needed from ulvan than UC hydrogel, which confirms the efficiency of adding chitosan to ulvan to improve its bioactivities.

Ulvan has been shown to have antimicrobial activity [60, 65]. Van Tran et al. [60] investigated the antimicrobial activity of ulvan obtained from *U. reticulate*, which demonstrated potent antimicrobial activity, with an inhibition zone diameter of 20 mm against *Enterobacter cloacae* and 18 mm against *E. coli*.

In contrast to Amin [66], who detected a high antimicrobial activity against human pathogenic bacteria and yeast such as *C. albicans*, *S. aureus*, *E. coli*, *P. aeruginosa*, and *B. subtilis* upon the use of a high concentration of ulvan (100 mg/mL), our results revealed that no antifungal activity was detected against the tested fungi. Recently, Gruskiene et al. [67] observed that using ulvan as a carrier with the antibacterial drug nisin increases the latter’s efficiency against Gram-positive bacteria compared to free nisin. Furthermore, ulvan possesses high antimicrobial activity against human, plant, poultry, fish, and animal pathogens, suggesting its potential use as a prebiotic in food processing [68] and as a preservative in packaged food and cosmetics, as well as in the pharmaceutical industry and medical applications against human pathogens [69].

On the contrary, after sterilization for 15 and 30 min at 121 °C and 1.5 Pa, the extracted ulvan completely lost its activity, indicating that it is thermolabile and susceptible to very high temperatures.

### Antibiotic Susceptibility of Indicator Pathogens

**Fig. 9:**
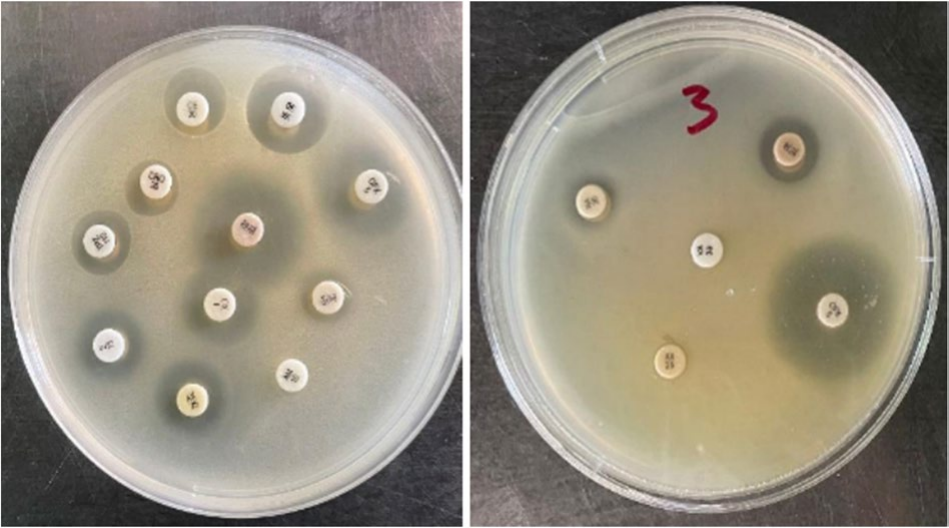
Antibiotic susceptibility of *S. aureus* ATCC 25,923 and *A. hydrophila* to different types of antibiotics using disc diffusion method

The antibiotic suitability of the indicator pathogens towards different types of antibiotics was evaluated for comparison. The data represented in Table 4 and Fig. 9 indicated that the clear zone diameters ranged from 18 mm against *S. aureus* to 30 mm against *K. pneumoniae*. In contrast, Tetracycline (TE)_30_, Ofloxacin (OFX)_5_, and Vancomycin (VA)_30_ exhibited more potent activity against some bacteria. The produced ulvan, or UC hydrogel, was more effective against tested pathogens than most tested antibiotics, which means that the extracted ulvan and UC hydrogel can be utilized as a natural alternative to antibiotics bypassing the adverse effects of antibiotic overuse and overcoming antibiotic resistance [44, 70].

**Table 4:** Antibiotic suitability test for bacterial pathogens towards different types of antibiotics

Antibiotic	Pathogen inhibition zone diameter (mm)							
	*B. Subtilis*	*E. coli*	*P. aeruginosa*	*K. pneumoniae*	*S. aureus*	*P. fluorescens*	*S. epidermidis*	*A. hydrophila*
Ampicillin (AM)_10_	5	-	5	-	-	-	-	-
Tetracycline (TE)_30_	11	24	13	25	21	10	-	23
Cephradine (CE)_30_	5	-	5	-	12	5	-	-
Nalidixic (NA)_30_	10	-	9	19	10	10	-	20
Amoxicillin (AX)_25_	5	20	6	14	15	6	-	-
Ofloxacin (OFX)_5_	27	26	27	30	18	27	10	20
Oxacillin (OX)_1_	5	-	5	-	15	-	-	-
Erythromycin (E)_15_	-	10	5	23	14	-	17	-
Ceftriaxone (CRO)_30_	5	-	5	-	11	5	-	-
Tazobactam (TPZ)_110_	5	-	6	-	13	-	-	-
Vancomycin (VA)_30_	6	27	6	26	17	10	7	12

### Anti-fouling Activity of the Purified Ulvan

**Fig. 10:**
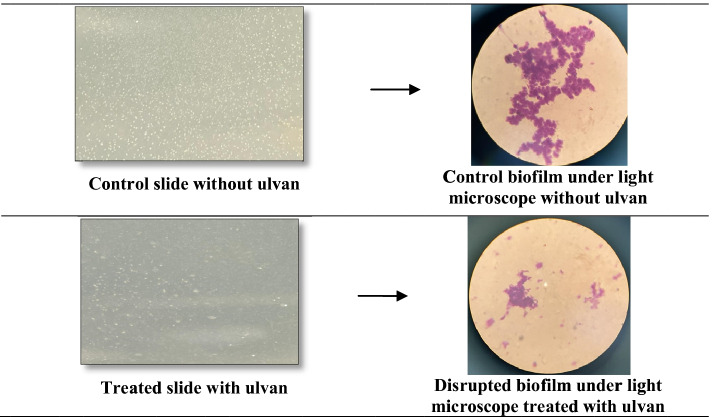
Anti-fouling activity of the purified ulvan. Macrographs show the biofilm under light microscope as dense collective (upper) in case of the control (without ulvan treatment), while the biofilm shown under light microscope as loose and disrupted (lower) when treated with ulvan

Seawater was used to test the anti-fouling characteristics of the purified ulvan from *U. lactuca.* The slides were examined directly and through a light microscope Fig. 10). The biofilm formed on the glass slide as a preliminary stage of fouling in the flasks treated with purified ulvan was weak and disrupted when compared to the control without any ulvan.

Many plants and seaweed natural compounds have antibiofilm and anti-fouling activity [71–73], making them promising and environmentally friendly alternatives to anti-fouling paint booster biocides now in use [74]. The electrostatic repulsions between the negative charges of the bacteria and the negatively charged carboxyl groups in the ulvan may illustrate the substantial reduction in bacterial adherence on the slide in the ulvan treated flask, which is consistent with the findings of Magnani et al. [75], who found that negative carboxyl group charges contributed to the reduction of *S. epidermidis* adherence. Similarly, Jeong et al. [11] found that the ulvan-grafted Ti/TiO_2_ surfaces had higher anti-fouling efficacy than the non-treated surface.

According to Gadenne et al. [10], the anti-adhesive capabilities of ulvans have been documented in the literature, but the factors influencing their anti-fouling properties have yet to be found. Furthermore, their findings revealed that the experimental immobilization conditions and the molecular weight of the polysaccharides resulted in distinct layer conformations that played a crucial role in the surface anti-adhesive properties.

On the contrary, this anti-fouling effect can also be attributed to the bactericidal effect of ulvan on fouling bacteria, as the bacterial count was detected quantitatively in both flasks. In addition, the data indicated a substantial reduction in the bacterial counts (about 57%), with the control flask having 3.0 × 10^4^ ± 0.07 CFU/mL, while the flask treated with ulvan having only 1.3 × 10^4^ ± 0.05 CFU/mL.

## Conclusion

The scientific interest in bio-based polymers is growing rapidly these days, and it is expected to continue to expand over time. In general, ulvan is one of the sulfated polysaccharides with unique features and qualities. Therefore, the current study focuses on the extraction of ulvan from *U. lactuca* with simple methodology. Collectively, our findings demonstrate that *U. lactuca* has a high potential in ulvan production. The chemical characterization of the semi-purified ulvan estimated polysaccharide content to be 36.50 g/100 g, with a high carbohydrate and sulfate contents 43.61% and 19.72%, respectively. In addition, the extracted ulvan showed considerable antimicrobial and anti-fouling activities, and the prepared UC hydrogel relatively improved the antibacterial activity and showed decreased MIC values. However, neither of them had any antifungal activity against tested fungi.

## Supplementary Information

Below is the link to the electronic supplementary material.

Supplementary file1 (DOCX 35 KB)

## Data Availability

All data are available upon request.
