# Supplementary material for: Considerable Production of Ulvan from Ulva lactuca with Special Emphasis on Its Antimicrobial and Anti-fouling Properties
Source: Appl Biochem Biotechnol. 2022 Mar 26;194(7):3097–118. doi: 10.1007/s12010-022-03867-y (PMC9205838; doi:10.1007/s12010-022-03867-y)
Supplement: Supplementary file 1 — Supplementary file1 (DOCX 35 KB) [file 12010_2022_3867_MOESM1_ESM.docx]

**Table S1** . Areas and average molecular weights (M.Wt) of the different polysaccharides of ulvan from *U.* lactuca

| **Distribution curve** | | **1** | **2** | **3** | **4** | **5** | **6** |
| --- | --- | --- | --- | --- | --- | --- | --- |
| **Elution time** | (RT ; min) | 24.31 | 25.95 | 28.60 | 33.39 | 38.85 | 41.87 |
| **Average** | (M.wt ; kDa) | 1.25E+05 | 8.39E+04 | 4.43E+04 | 1.40E+04 | 3.74E+03 | 1.81E+03 |
| **Area** | (%) | 4.27 | 16.35 | 46.12 | 29.50 | 1.41 | 2.35 |

**Table S2 .** The Gaussian distribution curves of the SEC chromatogram for the ulavn from *U.* lactuca after applying deconvolution method

| **Model** | **Gaussian** | | | | | |
| --- | --- | --- | --- | --- | --- | --- |
| Equation | y = y0 + A/(w*sqrt(pi/(4*ln(2)))) * exp(-4*ln(2)*(x-xc)^2/w^2) | | | | | |
| **Plot** | Molecular weight distribution curve 1 | Molecular weight distribution curve 2 | Molecular weight distribution curve 3 | Molecular weight distribution curve 4 | Molecular weight distribution curve 5 | Molecular weight distribution curve 6 |
| **y0** | 0.00409 ± 1.32799E-4 | 0.00409 ± 1.32799E-4 | 0.00409 ± 1.32799E-4 | 0.00409 ± 1.32799E-4 | 0.00409 ± 1.32799E-4 | 0.00409 ± 1.32799E-4 |
| **xc** | 24.31681 ± 0.00742 | 25.95948 ± 0.01509 | 28.60519 ± 0.02208 | 33.3952 ± 0.00269 | 38.85624 ± 0.02199 | 41.87628 ± 0.06137 |
| **A** | 0.40431 ± 0.02202 | 1.54863 ± 0.05998 | 4.36677 ± 0.04282 | 2.79332 ± 0 | 0.13349 ± 0.00617 | 0.22277 ± 0.00691 |
| **w** | 1.56394 ± 0.02515 | 2.85483 ± 0.05006 | 4.88138 ± 0.02325 | 4.35 ± 0 | 2.1153 ± 0.05274 | 4.14943 ± 0.1223 |
| Reduced Chi-Sqr | 7.36694E-5 | | | | | |
| **R-Square (COD)** | **0.99917** | | | | | |
| **Adj. R-Square** | **0.99917** | | | | | |
